# Multimodal MRI assessment for first episode psychosis: A major change in the thalamus and an efficient stratification of a subgroup

**DOI:** 10.1002/hbm.25276

**Published:** 2020-12-30

**Authors:** Andreia V. Faria, Yi Zhao, Chenfei Ye, Johnny Hsu, Kun Yang, Elizabeth Cifuentes, Lei Wang, Susumu Mori, Michael Miller, Brian Caffo, Akira Sawa

**Affiliations:** ^1^ Department of Radiology The Johns Hopkins University School of Medicine Baltimore Maryland USA; ^2^ Department of Biostatistics Indiana University, School of Medicine Indianapolis Indiana USA; ^3^ Department of Electronics and Information Harbin Institute of Technology Shenzhen Graduate School Guangdong China; ^4^ Department Psychiatry The Johns Hopkins University School of Medicine Baltimore Maryland USA; ^5^ Department of Psychiatry and Behavioral Sciences and Radiology Northwestern University Evanston Illinois USA; ^6^ Department of Biomedical Engineering The Whiting School of Engineering Baltimore Maryland USA; ^7^ Department of Biostatistics The Johns Hopkins University Bloomberg School of Public Health Baltimore Maryland USA; ^8^ Department of Neuroscience The Johns Hopkins University School of Medicine Baltimore Maryland USA; ^9^ Department of Mental Health The Johns Hopkins University Bloomberg School of Public Health Baltimore Maryland USA

**Keywords:** DTI, factor analysis, first‐episode psychosis, multimodal MRI, resting state fMRI, schizophrenia

## Abstract

Multi‐institutional brain imaging studies have emerged to resolve conflicting results among individual studies. However, adjusting multiple variables at the technical and cohort levels is challenging. Therefore, it is important to explore approaches that provide meaningful results from relatively small samples at institutional levels. We studied 87 first episode psychosis (FEP) patients and 62 healthy subjects by combining supervised integrated factor analysis (SIFA) with a novel pipeline for automated structure‐based analysis, an efficient and comprehensive method for dimensional data reduction that our group recently established. We integrated multiple MRI features (volume, DTI indices, resting state fMRI—rsfMRI) in the whole brain of each participant in an unbiased manner. The automated structure‐based analysis showed widespread DTI abnormalities in FEP and rs‐fMRI differences between FEP and healthy subjects mostly centered in thalamus. The combination of multiple modalities with SIFA was more efficient than the use of single modalities to stratify a subgroup of FEP (individuals with schizophrenia or schizoaffective disorder) that had more robust deficits from the overall FEP group. The information from multiple MRI modalities and analytical methods highlighted the thalamus as significantly abnormal in FEP. This study serves as a proof‐of‐concept for the potential of this methodology to reveal disease underpins and to stratify populations into more homogeneous sub‐groups.

## Graphical abstract

We combined automated structure‐based analysis with supervised integrated factor analysis to examine multiple MRI features (volume, DTI, resting state fMRI) in the whole brain of individuals with first‐episode psychosis. We successfully characterized this group, and subgroups with schizophrenia‐ and mood‐associated psychosis. We found robust abnormalities centered in the thalamus.

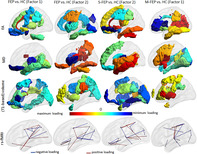

## 1 INTRODUCTION

Although neuroimaging abnormalities in patients with first episode psychosis (FEP) have been demonstrated, their quantitative distribution is still are under debate. Different patterns of atrophy in the frontal cortex, cingulate cortex, parahippocampal gyri, the basal ganglia, and the thalamus has been reported from multiple groups in the past (Buchy, Makowski, Malla, Joober, & Lepage, 2018; Calvo, Delvecchio, Altamura, Soares, & Brambilla, 2019; Castro‐de‐Araujo & Kanaan, 2017; Kuang et al., 2017; Makowski et al., 2019; Nakamura et al., 2007; Schubert, Clark, & Baune, 2015; Tordesillas‐Gutierrez et al., 2018). The inverse trend (gray matter increase) has also been reported (Dukart et al., 2017). Data in resting state functional MRI (rs‐fMRI) that compare FEP patients with healthy controls (HC) are also controversial, varying from no differences between groups to regional or diffuse differences (Alonso‐Solis et al., 2012; Argyelan et al., 2015; Bang et al., 2018; Choe et al., 2018; Ganella et al., 2018; Gohel et al., 2018; Huang et al., 2020; Tang et al., 2019). Previous observations of white matter changes have not been consistent. Differences in diffusion tensor imaging (DTI) were erratically reported in various brain areas such as the anterior limb of the internal capsule, the corpus callosum, the superior longitudinal fasciculus, and the uncinate. There was no specific cluster of white matter abnormalities that were unquestionably related to FEP (Deng et al., 2019; Di Biase et al., 2017; Kuswanto, Teh, Lee, & Sim, 2012; Lei et al., 2015; Ren et al., 2017; Serpa et al., 2017; Zhou et al., 2017). Recently, a large‐scale study from the ENIGMA group identified widespread white matter microstructural abnormalities in chronic schizophrenia (Kelly et al., 2017). The reproducibility of this finding in individuals with FEP is still unknown.

The inconsistency of imaging study findings in FEP can be attributed to several factors. These include the inconsistency among studies regarding the actual definition of FEP (Breitborde, Srihari, & Woods, 2009), the limited power to detect subtle abnormalities in small samples (Buchy et al., 2018; Emsley et al., 2017; Guma et al., 2017; Kong et al., 2011; Kuang et al., 2017; Lee et al., 2012; Lian et al., 2018; McNabb et al., 2018; Peters et al., 2008; Serpa et al., 2017), as well as the bias in the selection of MRI modalities and regions of interest (ROIs) (Baglivo et al., 2018; Cho et al., 2019; Forns‐Nadal et al., 2017; Huttlova et al., 2014; Lang et al., 2006; McHugo et al., 2018; Parellada et al., 2017; Sauras et al., 2017; Ublinskii et al., 2015; Vargas et al., 2018). Analyzing features through multiple MRI modalities over the whole brain became possible with the evolution of the scanners. Different neuroimaging modalities may capture different aspects of neuropathology and provide complementary information. The multimodal analysis reveals relationships between variables in imaging and nonimaging domains (e.g., genetics, cognition) and enables phenotypic characterization (Lerman‐Sinkoff, Kandala, Calhoun, Barch, & Mamah, 2019; Moser et al., 2018; Tognin et al., 2020). The combination of multiple observables has already proven to be valuable in conditions affecting multiple systems, from financial markets (Kim, Min, & Han, 2006; Lessmann, Baesens, Seow, & Thomas, 2015) to cancer (Kourou, Exarchos, Exarchos, Karamouzis, & Fotiadis, 2015), neurodegenerative diseases (Dai et al., 2012; Dyrba, Grothe, Kirste, & Teipel, 2015; Long et al., 2012; Zhang, Wang, Zhou, Yuan, & Shen, 2011), and psychosis (Schultz et al., 2012).

Schizophrenia is known to affect multiple domains (Fitzsimmons, Kubicki, & Shenton, 2013; Hirjak et al., 2019; Karlsgodt, Sun, & Cannon, 2010) and set up the ground for the initial attempts of multimodal analysis. (Aine et al., 2017; Calhoun & Sui, 2016; Meng et al., 2017; Shile et al., 2016; Sui, Huster, Yu, Segall, & Calhoun, 2014; Wang et al., 2015). Several recent studies have differentiated schizophrenia patients from HCs by combining data from functional and structural MRI (for a review, please see Rashid & Calhoun, 2020). They consistently found multimodal MRI classifiers more efficient than those based on single modalities (Cabral et al., 2016; Qureshi, Oh, Cho, Jo, & Lee, 2017; Yang, Liu, Sui, Pearlson, & Calhoun, 2010). While these studies focused on patients with established diagnosis of schizophrenia, mostly far from the clinical onset, this assessment was not fully applied to study patients in initial stages of illness, when this characterization is likely to have greater utility. Patients with FEP were mostly assessed in with limited number of ROIs and few MRI modalities (Deng et al., 2019; Keymer‐Gausset et al., 2018; Lei et al., 2015; Peruzzo, et al., 2015; Zhao et al., 2018).

Although its strengths, the implementation of this multimodal assessment is not straightforward: simply combining larger number of variables leads to multiple comparison issues and stress limitations of the sample size (Arbabshirani, Plis, Sui, & Calhoun, 2017). Multi‐institutional brain imaging studies have recently emerged to overcome conflicting results among individual studies (Thompson et al., 2020). However, adjusting multiple variables at the technical and cohort levels remains a continuous challenge (Levin‐Schwartz, Calhoun, & Adali, 2017). Developing strategies to reduce the dimensions of data, while preserving the information is a field in current development (Bassett, Xia, & Satterthwaite, 2018; Lottman et al., 2018; Miller, Vergara, & Calhoun, 2018; Qi et al., 2019; Sui, Adali, Yu, Chen, & Calhoun, 2012; Tu et al., 2019; Xia et al., 2018). A strong basis on biological knowledge is needed to develop and implement the algorithms in a comprehensive and practical way, so the research can eventually be translated to clinical field.

In order to overcome these challenges, we analyzed multiple MRI characteristics in the FEP and HC groups through whole‐brain automated segmentation in a fully data‐driven integrative approach that we have recently established (Miller, Faria, Oishi, & Mori, 2013; Rezende et al., 2019). This approach aims to reduce the dimensions of image data in a biologically meaningful way, increasing the statistical power and offering comprehensive results about the brain structure (Faria, Liang, Miller, & Mori, 2017; Miller et al., 2013; Mori, Oishi, Faria, & Miller, 2013). We combined this approach with supervised integrated factor analysis (SIFA) (Li & Jung, 2017) to examine multiple MRI features (volume, DTI indices, rs‐fMRI) in the whole brain of FEP participants. We also accessed whether this multimodal approach would be efficient on classification of participants in subgroups of individuals with schizophrenia and schizoaffective disorder (S‐FEP) and individuals with bipolar disorder and major depressive disorder with psychotic features (M‐FEP). We investigated how this novel analytical pipeline may provide evidence of pathological abnormalities in the early stage of illness and potentially aid to stratify groups of clinical relevance.

## 2 MATERIALS AND METHODS

### 2.1 Recruitment and participants

This study was approved and conducted using guidelines established by the Johns Hopkins School of Medicine Institutional Review Board and in accordance with The Code of Ethics of the World Medical Association (1964 Declaration of Helsinki). Each participant received a full explanation of the study procedures. Written informed consent was obtained for all participants 18 years of age and older. Parental consent and assent was obtained for all participants under 18 years of age. HCs and FEP patients, with FEP being defined as those who had experienced their first episode of psychosis within the 2 years prior to their enrollment, were recruited through the Johns Hopkins Schizophrenia Center. Details about the recruitment, inclusion and exclusion criteria, demographics, and clinical features are described in published articles by our group (Kamath et al., 2019; Kamath, Lasutschinkow, Ishizuka, & Sawa, 2018; Wang et al., 2019). In the present study, the participants included individuals with FEP (*n* = 87) [SZ (*n* = 47), schizoaffective disorder (*n* = 14), bipolar disorder with psychotic features (*n* = 20), major depressive disorder with psychotic features (*n* = 6)] and 62 HC. We included individuals with schizophrenia and schizoaffective disorder in the schizophrenia‐associated psychosis group (S‐FEP) and individuals with bipolar disorder with psychotic features and major depressive disorder with psychotic features in the mood‐associated psychosis group (M‐FEP). This decision was based on two meta‐analyses (Pagel, Baldessarini, Franklin, & Baethge, 2013; Rink, Pagel, Franklin, & Baethge, 2016) that found patients with schizoaffective to have illness characteristics that align more closely with patients with schizophrenia than with those with bipolar disorder and major depression.

### 2.2 Imaging

The multimodal MRI was performed on a 3 T scanner, and included T1 high‐resolution‐weighted images (T1‐WI), diffusion weighted images (DWI), and resting state functional MRI (rs‐fMRI). The image parameters were: (a) T1‐WI: sagittal orientation, original matrix 170 × 170, 256 slices, voxel size 1 × 1 × 1.2 mm, TR/TE 6700/3.1 ms; (b) DWI: axial orientation, original matrix 128 × 128, 70 slices, voxel size 0.83 × 0.83 × 2.2 mm, TR/TE 8500/61 ms, 32 gradients, b factor 1,000 s/mm^2^; and (c) rs‐fMRI: axial orientation, original matrix 80 × 80, 36 slices, voxel size 3 × 3 × 4 mm, TR/TE 2000/30 ms, 210 time points.

**FIGURE 1:**
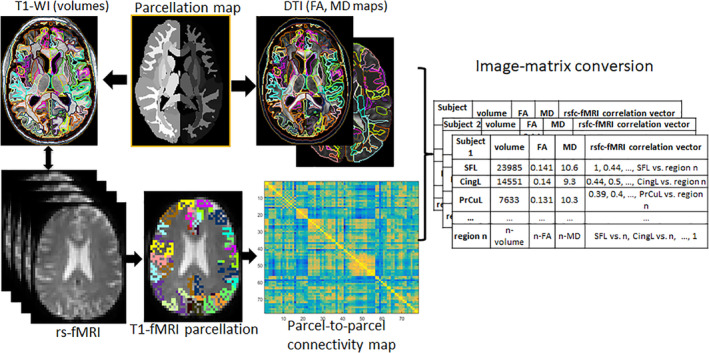
Schematic representation of the automated image parcellation using MRICloud (www.MRICloud.org). Each brain image is mapped to a set of multiple atlases and the pre‐defined labels are applied to each original brain. T1‐weighted images (for volumetric analysis) and DTI pipelines run in parallel. For the low‐resolution modalities (e.g., rs‐fMRI), the labels are brought to the original space by co‐registering the T1‐WIs. Through this process, the multiple MRI modalities are converted to a matrix of structures by image features, which represent each individual

We analyzed multiple MRI contrasts in an atlas‐based, structurally focused, integrative, and non‐biased whole‐brain approach (Figure 1). The images were automatically segmented and postprocessed through MRICloud (www.MRICloud.org) (Mori et al., 2016), a public web‐based service for multi‐contrast imaging segmentation and quantification. In MRICloud, the process for segmenting the T1‐WI, used for volumetric analysis, involves: (a) orientation and homogeneity correction, (b) two‐level brain segmentation, (c) image mapping based on a sequence of linear algorithms and Large Deformation Diffeomorphic Mapping (LDDMM), and (d) a final step of multi‐atlas labeling fusion (MALF), adjusted by PICSL (Tang et al., 2013). For the DWI postprocessing, the tensor reconstruction and quality control follows the algorithm used by DtiStudio (www.MRIStudio.org). The automated DTI segmentation is similar to that used for T1‐WIs and differs in the use of complementary contrasts (mean diffusivity [MD], fractional anisotropy [FA], and eigenvector such as fiber orientation) and a diffeomorphic likelihood fusion algorithm (Tang et al., 2014) for multi‐atlas mapping.

For the rs‐fMRI postprocessing (Faria et al., 2012), the T1‐WI and its respective segmentations are co‐registered to the motion and slice timing‐corrected resting‐state time points. Intensity and motion “outliers” are extracted with ART (SPM toolbox). Time courses are extracted from all the cortical and subcortical gray matter regions defined in the atlases and regressed for physiological nuisance. For “denoising” the time courses, we used the six motion parameters as regressors, as well as CompCor (Behzadi, Restom, Liau, & Liu, 2007) to regress nonneuronal physiological activity. These procedures, automatically performed in MRICloud, are similar to those adopted by major rs‐fMRI postprocessing pipelines (e.g., SPM) and are detailed described in our previous publication (Faria et al., 2012). Furthermore, we calculated frame‐wise displacement (FD) using six motion parameters (Power, Barnes, Snyder, Schlaggar, & Petersen, 2012). No participant had FD > 0.5, and only one participant had FD > 0.3. Both groups had small average values of head motion (mean FD < 0.1). Still, the FEP group showed larger FD than HCs (*p* = .02), justifying our procedure of using the motion‐corrected time courses for the analysis. Seed‐by‐seed correlation matrices are obtained from the “nuisance‐corrected” time courses and z‐transformed by the Fisher's method. Note that MRICloud pipelines include well accepted protocols to minimize artifacts, as those created by motion, in DWI and rsfMRI. These and other technical procedures are detailed in the original publications (Faria et al., 2012; Jiang, van Zijl, Kim, Pearlson, & Mori, 2006; Tang et al., 2014).

After the multimodal brain segmentation and quantification, each individual's brain was represented by a vector of image features: (a) volumes of 198 structures automatically segmented from T1‐WIs, (b) fractional anisotropy (FA) and (c) mean diffusivity (MD) means of 96 structures automatically segmented from DTIs, and (d) 2415 pairwise resting‐state z‐correlations from 70 seeds in the superficial gray matter (i.e., cortex) and the deep gray matter (i.e., basal ganglia plus thalamus). This process is represented in Figure 1.

### 2.3 Statistical analyses

We investigated group differences between FEP groups and HC (HC vs. FEP, HC vs. S‐FEP, and HC vs. M‐FEP) in the various imaging modalities by using two‐sample *t* tests. The False Discovery Ratio (FDR) (Benjamini & Hochberg, 1995) was used to correct for multiple comparisons at a “*q*” (“*p*‐corrected”) level of significance <.05.

The SIFA was implemented to integrate data collected from multiple imaging modalities while facilitating characterization with auxiliary covariates. For each modality, it is assumed that the data consist of two types of latent factors: (a) common factors shared across all modalities and (b) individual factors specific to the data source. For both types of latent factors, a linear regression model of the covariates was used to capture the influential effects. Since the goal of implementing SIFA is to integrate multiple imaging modalities and identify joint structure, we only present the results of the estimated common factors. The latent factors are assumed to be independent, which is analogous to the assumption imposed in the independent component analysis (ICA) (McKeown & Sejnowski, 1998) widely used in neuroimaging analysis. The ranks of the factors were chosen based on a likelihood cross validation (LCV) approach. Similarly to what is done in a sparse principal components analysis (Zou, Hastie, & Tibshirani, 2006), loading profiles were sparsified through a penalized regression to achieve the purpose of feature selection. The covariates for adjustment included group (FEP, HC, S‐FEP, M‐FEP) as well as race, sex, and age. Because in our study the data dimension is slightly unbalanced among modalities, we implemented the “SIFA‐B” approach (Li & Jung, 2017), which is robust to unbalanced dimensions due to orthogonal and equal norm identifiability conditions. To test the significance of the coefficients in the regression models, confidence intervals were obtained using 500 bootstrap samples. Details about SIFA, the procedure to draw inference, and the classification approach are in Section A of the Supplementary material.

While group features may reveal pathological mechanisms, it is important to know if the multimodal features revealed by SIFA are expressed at an individual level. Therefore, we investigated the power of these image features to classify individuals within their respective diagnostic groups. It is also important to detect the effectiveness of models using multiple modality features, as compared with those using singular modality features for individual classification. For this purpose, we used leave‐one‐out cross‐validated receiver operating characteristic (ROC) curves. The logistic classification models were trained using the latent factors estimated from the factor analysis. The area under the curve (AUC) and the 95% confidence interval were calculated. We also calculated the sensitivity, specificity, and F1 score of the classification performance using the latent factors estimated from SIFA. We compared the performance with the support vector machine (SVM) approach considering three different types of kernel functions: linear, polynomial and radial kernels. For all approaches, five sets of data were considered to train the prediction model: (a) data of all modalities, (b) rs‐fMRI data, (c) FA (from DTI), (d) MD (from DTI), and (e) T1‐volumetric data. The data analyzed in this study and the analytical code are available under request to the authors.

## 3 RESULTS

### 3.1 Demographic analysis

Clinical and demographic variables are presented in Table 1. Since covariates were group‐matched based on study design, the FEP group, its subgroups, and the HC group did not differ with respect to age, sex, race, and parental education. The FEP subgroups did not differ in illness duration and antipsychotic medication dosage.

**TABLE 1:** Demographic and clinical characteristics

	Mean ± *SD* [min max]				*p*‐value (T or *χ* ^2^ statistic)			
	HC (*n* = 62)	All FEP (*n* = 87)	S‐FEP (*n* = 61)	M‐FEP (*n* = 26)	HC vs. FEP	HC vs. S‐FEP	HC vs. M‐FEP	S‐FEP vs. M‐FEP
Age (years)	23.4 ± 3.6 [16 33]	22.5 ± 4.3 [15 35]	22.3 ± 4.2 [15 34]	23 ± 4.8 [17 35]	0.16 (−1.4)	0.16 (−1.5)	0.64 (−0.46)	0.57 (−0.57)
Sex (M:F)	43:19	53:14	49:12	14:12	0.82 (0.04)	0.82 (1.4)	0.25 (1.3)	0.02 (5.1)
Race (aa:c:as:h:o)	33:23:2:2:2	43:34:4:3:3	33:23:2:1:2	10:11:2:2:1	0.85 (0.03)	0.85 (1)	0.52 (0.39)	0.52 (0.4)
Parental education (years)	15.18 (2.62)	15.1 ± 3.2	14.96 (3.06)	15.42 (2.63)	0.84 (0.03)	0.51 (0.4)	0.73 (0.25)	0.65 (0.36)
Age at onset (years)			21.3 ± 4.1 [14 32]	22.3 ± 4.7 [15 34]				0.35 (−0.92)
Illness duration (months)			13.2 (8.9)	10.8 (9.5)				0.3 (1)
Antipsychotic dose^a^			381.9 ± 302.9	267.3 ± 221.5				0.07 (1.8)
SAPS			3.9 ± 3.7 [0 15]	2.2 ± 3.7 [0 12]				0.06 (1.9)
SANS			8.5 ± 5.0 [0 20]	4.4 ± 4.4 [0 16]				0.0004 (3.7)

*Note:* Race codes: aa, African American; as, Asian; c, Caucasian; h, Hispanic; o: others.

Abbreviations: HC, healthy controls; M‐FEP, major depression and bipolar disorder with psychiatric features; SANS, Scale for the Assessment of Negative Symptoms; SAPS, scale for the assessment of positive symptoms; S‐FEP, schizophrenia and schizoaffective disorders.

^a^ Antipsychotic medication dosages were converted to chlorpromazine equivalents using published reference tables (Woods SW. Calculation of CPZ Equivalents. In: Equivalent C, ed; 2005). Medication dosage information was unavailable for six patients.

### 3.2 Group comparison (FEP vs. HC) of imaging characteristics in each modality

**FIGURE 2:**
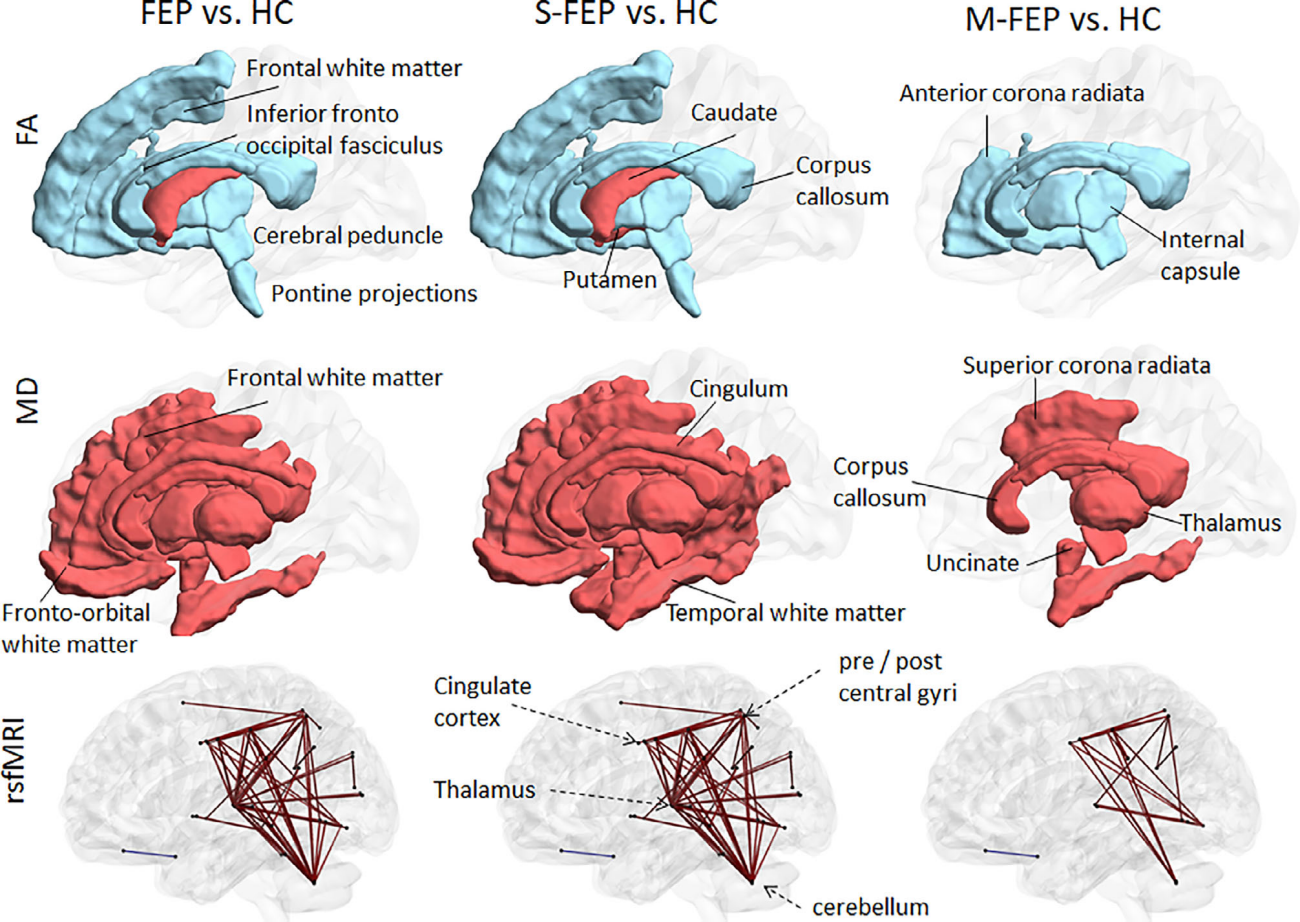
Differences in imaging features between groups. Regions with abnormal DTI indices [FA (top row), MD (middle row)] and edges of abnormal rs‐fMRI synchrony (bottom row) in FEP (left column), S‐FEP (middle column), and M‐FEP (right column) compared with HC. Blue are lower mean values in FEP groups compared with controls; red are higher mean values in FEP compared with controls. Visualization with the BrainNet Viewer (http://www.nitrc.org/projects/bnv/, by Xia, Wang, & He, 2013)

No volumetric differences were found between FEP and HC groups after the multiple comparisons correction. With respect to DTI differences, FEP individuals showed lower FA in the global white matter (defined as the average of all white matter segments) as compared with HC. More specifically, FEP and HC groups differed in DTI indices (FA and MD) in most subsegments of the projection fibers (cortico‐spinal and spino‐cortical). These fibers represent most of the motor and sensorial tracts. These two groups also differed in MD and FA at the main commissural fibers, as represented by the corpus callosum (Figure 2, Table 2). Association areas also showed abnormal DTI indices. Compared with the HC group, the FEP group showed lower FA and higher MD in the corona radiata and the inferior fronto‐occipital fasciculus; lower FA in the white matter beneath the right superior frontal gyrus; as well as higher MD in the uncinate fasciculus, cingullum, and in the white matter beneath the inferior temporal and middle and inferior frontal gyri. In the deep nuclei, FEP showed lower FA in the globus pallidus, higher FA in the caudate, and higher MD in the thalamus and the putamen when compared with HC.

**TABLE 2:** Group differences

		Group means				HC vs. FEP		HC vs. S‐FEP		HC vs. M‐FEP	
		HC	FEP	S‐FEP	M‐FEP	T	*p*‐value	T	*p*‐value	T	*p*‐value
**Fractional anisotropy—FA**											
Projection	Cerebral peduncle	0.670	0.653	0.654	0.649	−6.065	.000	−4.853	.000	−5.773	.000
	Pontine projections	0.552	0.539	0.536	0.545	−4.310	.000	−4.595	.000	−1.747	.084
	Post limb int capsule	0.636	0.626	0.626	0.628	−3.555	.000	−3.409	.001	−2.097	.039
	Ant limb int capsule	0.570	0.559	0.560	0.556	−3.894	.000	−3.234	.001	−3.066	.003
	Corpus callosum	0.610	0.593	0.597	0.585	−5.715	.000	−3.900	.000	−7.694	.000
Assoc.	Inf frontoccipital fasc	0.445	0.435	0.438	0.430	−2.958	.003	−2.050	.042	−2.842	.006
	Ant corona radiata	0.433	0.427	0.428	0.425	−2.334	.020	−1.731	.045	−2.228	.029
	Sup frontal WM	0.384	0.381	0.380	0.381	−2.114	.035	−2.153	.032	−1.130	.261
Nucleae	Caudate	0.220	0.233	0.236	0.223	3.633	.000	3.881	.000	0.825	.412
	Globus pallidus	0.378	0.363	0.362	0.364	−2.221	.027	−2.184	.030	−1.341	.183
	Putamen	0.230	0.238	0.239	0.233	1.881	.061	2.040	.043	0.551	.583
	Total white matter	0.456	0.452	0.453	0.450	−2.863	.005	−1.999	.047	−2.924	.005
**Mean diffusivity—MD (× 10^−4^, in mm^2^/s)**											
Project.	Cerebral peduncle	8.707	8.932	8.922	8.956	3.910	.000	3.424	.001	3.083	.003
	Ant limb int capsule	7.553	7.713	7.748	7.631	3.330	.001	3.717	.000	1.077	.285
	Post limb int capsule	7.200	7.313	7.307	7.325	2.700	.007	2.386	.018	2.041	.044
	Corpus callosum	9.280	9.552	9.562	9.528	5.444	.000	5.400	.000	2.962	.004
Association	Sup corona radiata	7.340	7.462	7.473	7.436	3.710	.000	3.603	.000	2.213	.029
	Ant corona radiata	8.441	8.562	8.588	8.501	3.022	.003	3.330	.001	1.047	.298
	Inf frontoccipital fasc	8.402	8.573	8.601	8.509	3.200	.002	3.323	.001	1.521	.131
	Cingullum	7.747	7.847	7.857	7.823	2.451	.015	2.506	.013	1.316	.191
	Uncinate	8.580	8.716	8.717	8.712	2.913	.004	2.669	.008	2.192	.030
	Middle fronto‐orbital	9.614	9.779	9.838	9.639	2.166	.031	2.582	.010	0.242	.809
	Middle frontal WM	8.323	8.408	8.425	8.367	2.580	.010	2.911	.004	0.920	.360
	Inferior frontal WM	8.198	8.281	8.304	8.225	2.157	.032	2.527	.012	0.514	.608
	Middle temporal WM	8.512	8.577	8.599	8.525	1.781	.076	2.236	.026	0.256	.798
	Inferior temporal WM	8.752	8.940	8.934	8.953	3.964	.000	3.432	.001	3.157	.002
Nuc.	Thalamus	8.360	8.505	8.501	8.514	3.065	.002	2.743	.007	2.246	.027
	Putamen	7.184	7.349	7.383	7.271	3.638	.000	3.974	.000	1.432	.155
**Resting state fMRI**											
	PoCGL_CerebellumL	0.339	0.529	0.565	0.446	4.279	.000	4.364	.000	2.178	.033
	PoCGL_CerebellumR	0.362	0.560	0.603	0.460	4.401	.000	4.575	.000	2.048	.045
	SPGL_ThalamusR	0.102	0.299	0.322	0.245	4.350	.000	4.456	.000	2.119	.040
	SPGR_ThalamusR	0.132	0.309	0.344	0.227	4.092	.000	4.401	.000	1.617	.113
	PrCGL_CerebellumR	0.402	0.588	0.619	0.513	4.188	.000	4.289	.000	2.023	.048
	PrCGL_CerebellumL	0.360	0.541	0.567	0.481	4.131	.000	4.109	.000	2.176	.035
	PrCGL_ThalamusR	0.262	0.449	0.479	0.379	4.014	.000	4.138	.000	1.808	.078
	MTGR_ThalamusR	0.133	0.321	0.361	0.227	3.729	.000	4.121	.000	1.389	.171
	PoCGR_CerebellumR	0.355	0.545	0.565	0.499	4.253	.000	4.051	.000	2.495	.016
	SOGL_CuR	0.916	1.064	1.102	0.975	3.480	.001	4.005	.000	0.976	.334
	IOGR_ThalamusR	0.102	0.282	0.302	0.235	4.042	.000	4.014	.000	2.251	.029
	SPGL_GPR	0.114	0.237	0.273	0.154	3.319	.001	3.958	.000	0.784	.437
	SFGR_SPGL	0.335	0.504	0.523	0.460	3.361	.001	3.463	.001	1.824	.074
	MFOGR_STGR_pole	0.387	0.236	0.236	0.238	−3.914	.000	−3.608	.000	−2.911	.005
	PoCGL_SMGL	0.488	0.675	0.685	0.652	3.775	.000	3.469	.001	2.826	.006
	PoCGL_SMGR	0.538	0.680	0.711	0.606	3.107	.002	3.479	.001	1.204	.233
	PoCGL_IOGR	0.349	0.530	0.548	0.487	3.526	.001	3.495	.001	2.046	.046
	PoCGL_ThalamusR	0.229	0.414	0.439	0.357	3.769	.000	3.824	.000	1.831	.074
	PoCGR_PrCGL	1.020	1.193	1.219	1.132	3.292	.001	3.549	.001	1.705	.093
	PoCGR_SPGL	0.352	0.590	0.593	0.583	4.057	.000	3.629	.000	3.368	.001
	PoCGR_ThalamusL	0.217	0.370	0.399	0.304	3.156	.002	3.500	.001	1.206	.234
	PoCGR_ThalamusR	0.252	0.425	0.456	0.354	3.501	.001	3.733	.000	1.472	.148
	PrCGL_SPGL	0.435	0.638	0.643	0.627	3.949	.000	3.507	.001	3.127	.003
	PrCGL_SPGR	0.447	0.650	0.669	0.605	3.774	.000	3.642	.000	2.680	.009
	PrCGL_IOGR	0.337	0.532	0.537	0.520	3.972	.000	3.684	.000	2.628	.012
	PrCGL_ThalamusL	0.246	0.417	0.441	0.361	3.601	.000	3.722	.000	1.765	.084
	PrCGR_SPGL	0.442	0.651	0.653	0.645	4.003	.000	3.550	.001	3.365	.001
	PrCGR_IOGR	0.386	0.559	0.566	0.543	3.787	.000	3.535	.001	2.524	.015
	PrCGR_CerebellumL	0.425	0.580	0.606	0.518	3.457	.001	3.545	.001	1.694	.096
	PrCGR_CerebellumR	0.376	0.553	0.579	0.492	3.819	.000	3.815	.000	2.154	.035
	PrCGR_ThalamusR	0.271	0.450	0.480	0.380	3.568	.000	3.845	.000	1.489	.144
	SPGL_ThalamusL	0.124	0.277	0.294	0.236	3.324	.001	3.477	.001	1.654	.105
	SPGR_dorsal_ACCL	0.371	0.543	0.589	0.436	3.255	.001	3.732	.000	1.035	.305
	SPGR_dorsal_ACCR	0.417	0.570	0.612	0.473	3.156	.002	3.566	.001	0.996	.323
	SPGR_CerebellumR	0.370	0.532	0.570	0.444	3.494	.001	3.828	.000	1.446	.153
	SPGR_ThalamusL	0.117	0.271	0.301	0.199	3.446	.001	3.769	.000	1.366	.178
	SMGL_CerebellumL	0.253	0.409	0.441	0.333	3.505	.001	3.724	.000	1.401	.168
	SMGR_ThalamusR	0.163	0.323	0.356	0.246	3.329	.001	3.525	.001	1.492	.141
	AGL_PCCR	0.443	0.612	0.609	0.621	3.949	.000	3.486	.001	2.792	.008
	STGL_CerebellumL	0.315	0.448	0.491	0.346	2.929	.004	3.514	.001	0.535	.595
	STGL_ThalamusR	0.277	0.461	0.493	0.388	3.351	.001	3.530	.001	1.492	.142
	STGR_CerebellumL	0.307	0.454	0.489	0.374	3.439	.001	3.816	.000	1.169	.248
	STGR_ThalamusR	0.276	0.452	0.488	0.368	3.241	.001	3.587	.000	1.164	.251
	MTGL_PCCL	0.522	0.640	0.676	0.556	2.987	.003	3.696	.000	0.567	.573
	MTGL_ThalamusL	0.159	0.308	0.342	0.228	3.118	.002	3.569	.001	0.968	.339
	MTGL_ThalamusR	0.135	0.286	0.329	0.186	3.045	.003	3.633	.000	0.683	.498
	MTGR_PCCL	0.470	0.597	0.621	0.542	3.247	.001	3.496	.001	1.356	.181
	MTGR_CerebellumL	0.380	0.504	0.541	0.418	2.967	.004	3.496	.001	0.658	.514
	MTGR_PutR	0.127	0.272	0.307	0.188	3.211	.002	3.667	.000	1.068	.290
	MTGR_ThalamusL	0.127	0.294	0.327	0.216	3.433	.001	3.739	.000	1.361	.180
	FuGL_ThalamusR	0.133	0.275	0.301	0.214	3.250	.001	3.478	.001	1.352	.183
	SOGL_ThalamusR	0.121	0.259	0.283	0.203	3.352	.001	3.535	.001	1.464	.150
	SOGR_ThalamusR	0.116	0.251	0.268	0.211	3.284	.001	3.501	.001	1.560	.126
	MOGL_ThalamusR	0.174	0.318	0.358	0.224	3.287	.001	3.859	.000	0.796	.430
	MOGR_ThalamusR	0.156	0.299	0.325	0.236	3.350	.001	3.682	.000	1.260	.215
	IOGL_ThalamusR	0.112	0.261	0.284	0.208	3.327	.001	3.472	.001	1.474	.148
	IOGR_LGL	0.412	0.614	0.633	0.570	3.614	.000	3.539	.001	2.190	.033
	IOGR_CerebellumL	0.450	0.589	0.608	0.545	3.424	.001	3.488	.001	1.781	.081
	LGL_CerebellumL	0.471	0.600	0.627	0.537	3.347	.001	3.641	.000	1.344	.185
	LGR_CerebellumL	0.426	0.564	0.587	0.511	3.621	.000	3.745	.000	1.764	.084
	LGR_CerebellumR	0.415	0.557	0.575	0.516	3.556	.001	3.553	.001	1.919	.061
	LGR_ThalamusR	0.178	0.341	0.345	0.333	3.781	.000	3.491	.001	2.604	.012
	SPGR_CerebellumL	0.418	0.562	0.596	0.482	3.105	.002	3.445	.001	1.146	.256
	MTGR_CerebellumR	0.375	0.500	0.535	0.419	2.951	.004	3.454	.001	0.737	.465
	MTGR_PutL	0.116	0.252	0.281	0.185	3.154	.002	3.448	.001	1.381	.172
	PoCGL_SPGL	0.460	0.671	0.679	0.653	3.735	.000	3.418	.001	2.661	.010
	PoCGL_SPGR	0.516	0.702	0.724	0.650	3.408	.001	3.417	.001	2.182	.032
	IOGR_LGR	0.418	0.617	0.634	0.575	3.511	.001	3.415	.001	2.189	.033
	STGR_CerebellumR	0.308	0.446	0.479	0.367	3.067	.003	3.405	.001	1.030	.308
	SMGL_CerebellumR	0.286	0.419	0.452	0.341	3.035	.003	3.397	.001	0.983	.330
	LGL_CerebellumR	0.444	0.571	0.595	0.515	3.145	.002	3.395	.001	1.344	.185
	SPGR_PrCuL	0.418	0.590	0.615	0.532	3.212	.002	3.389	.001	1.626	.110
	PoCGL_ThalamusL	0.215	0.373	0.397	0.317	3.184	.002	3.373	.001	1.442	.156
	STGL_CerebellumR	0.327	0.452	0.499	0.342	2.716	.007	3.375	.001	0.257	.798
	PrCGR_ThalamusL	0.231	0.388	0.413	0.328	3.072	.003	3.327	.001	1.336	.188
	SPGL_LGR	0.271	0.451	0.456	0.442	3.663	.000	3.330	.001	2.713	.009
	FuGR_ThalamusR	0.132	0.267	0.292	0.209	3.092	.002	3.338	.001	1.295	.201
	IOGR_ThalamusL	0.101	0.257	0.266	0.235	3.503	.001	3.336	.001	2.273	.027
	PoCGR_SMGL	0.402	0.588	0.592	0.580	3.574	.001	3.314	.001	2.683	.009
	PoCGR_CerebellumL	0.402	0.550	0.570	0.505	3.348	.001	3.299	.001	1.740	.089
	SPGL_dorsal_ACCL	0.389	0.528	0.567	0.437	2.842	.005	3.284	.001	0.751	.457
	SPGL_PCCR	0.154	0.325	0.330	0.314	3.479	.001	3.286	.001	2.182	.035
	SPGL_CerebellumR	0.420	0.568	0.593	0.509	3.067	.003	3.274	.001	1.531	.131

*Note:* For the DTI analysis of FA and MD, the white matter is categorized in projection, association, and commissural (corpus callosum) fibers. We also analyzed deep nucleae and the white matter as a whole (“total white matter”). *p*‐value of 0 indicates *p*‐value < .0001.

Abbreviations: L: left, R: right; post: posterior, ant: anterior, sup: superior, inf: inferior, int: internal, fasc: fasciculus, WM: white matter. PoCG, PrCG: postcentral, precentral gyrus; SFG: superior frontal; MFOG: middle frontorbital gyrus; SPG: superior parietal gyrus; MTG, STG: middle, inferior temporal gyrus; SOG, IOG, MOG: superior, inferior, middle occipital gyrus; SMG: supramarginal gyrus; AG: angular gyrus; Fu: fusiform gyrus; CU: cuneus gyrus; LG: lingual gyrus; ACC, PCC: anterior, posterior cingulate cortex; GP: globus pallidus; Put: putamen.

With respect to rs‐fMRI differences, the FEP group showed higher rs‐fMRI z‐correlations than the HC group between several pairs of regions. Regions most often detected as seeds of abnormal correlations were the thalamus, the cerebellum, the somato‐sensorial cortex (parietal, post‐ and pre‐central), and the cingulate cortex (Figure 2, Table 2).

### 3.3 Subgroup comparison (S‐FEP and M‐FEP vs. HC) of imaging characteristics in each modality

Given that the FEP group and the HC group display anatomical differences, we next addressed the possible FEP subgroup that might contribute most to these differences. As described in the Methods section, we subdivided the FEP subjects into S‐FEP and M‐FEP groups and compared these individual groups with the HC group.

The differences detected between S‐FEP and HC were more widespread and had a higher effect size than those detected between FEP and HC. This is of great importance, considering the fact that S‐FEP is a subset, and therefore a smaller group than the more general FEP group. Volumetric differences did not overcome the threshold for multiple comparison correction, possibly due to the inclusion of individuals in early disease stage, whose brain structure was under minimum effect of the treatment and disease chronicity (van Erp et al., 2016; Vita, De Peri, Deste, Barlati, & Sacchetti, 2015). Yet, the S‐FEP group tended to have a larger Sylvian fissure (*p =* .028) and cingulum sulci (*p =* .027) than HC. This indirectly indicated possible atrophy or anatomical abnormalities of the adjacent structures (planum temporalis, insula, and cingullum), a progressive feature of psychotic brains (Kasai et al., 2003; Lee et al., 2016; Rosa et al., 2015). In addition to what was observed in the FEP versus HC comparison, the DTI abnormalities spread to the putamen and the white matter beneath middle temporal (Figure 2, Table 2). The rs‐fMRI abnormalities had, in general, a higher effect size than in the FEP versus HC comparison (Table 2).

No differences in volume were detected between M‐FEP and HC. Differences in DTI and rs‐fMRI between M‐FEP and HC were more constrained to a few regions and had a lower effect‐size than those observed between S‐FEP and HC (Figure 2, Table 2).

### 3.4 Multimodal characterization (SIFA) of FEP group

Given that the group comparison in the present study still involved many features from multiple MRI modalities (hundreds of volumes and DTI indices, and thousands of rs‐fMRI), we next applied SIFA for data integration. As described in the Materials and Methods section, SIFA allows us to identify the latent factors (i.e., the combination of features) related to the different groups, as well as leverage information across modalities.

**FIGURE 3:**
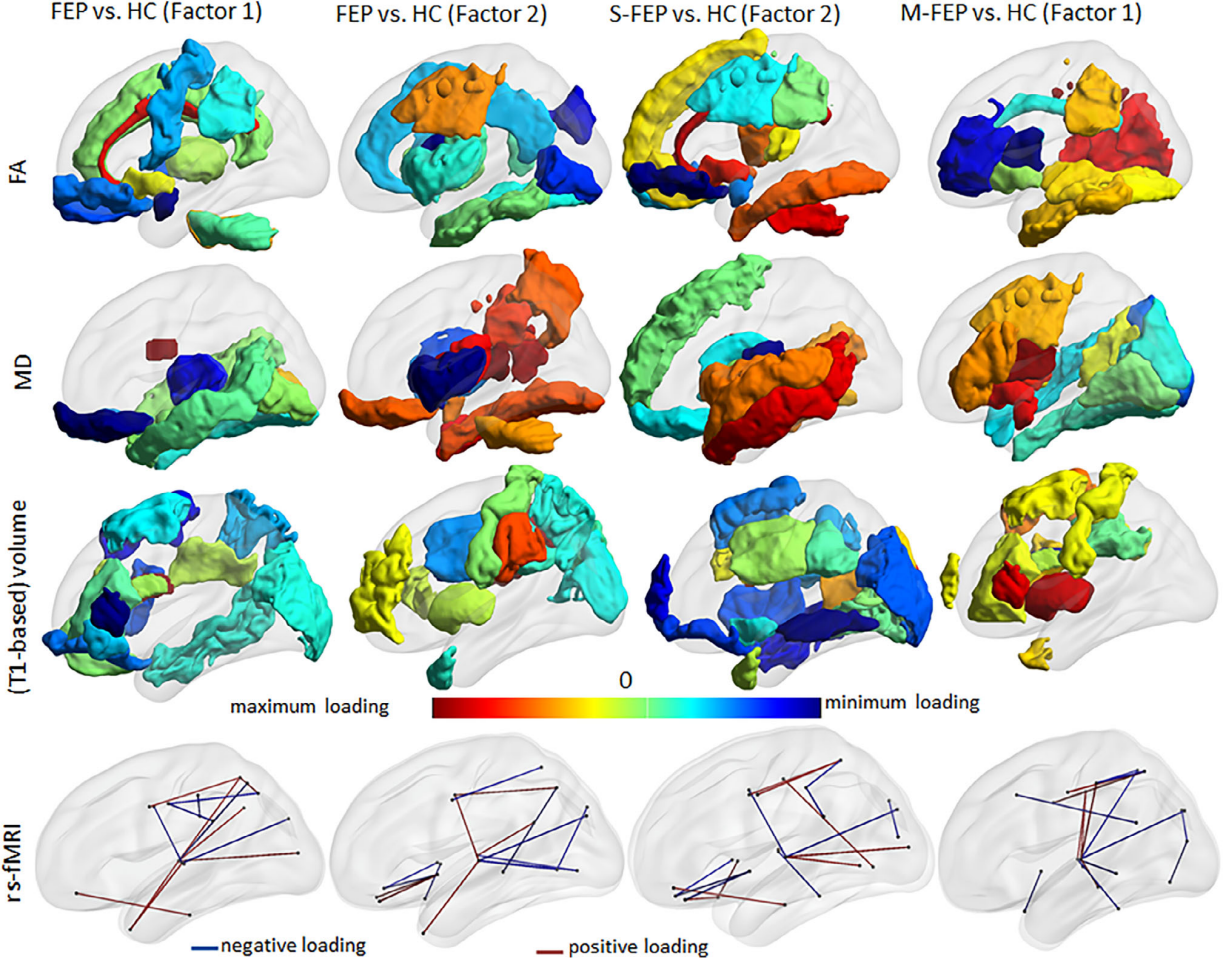
Characterization of FEP group and subgroups (S‐FEP and M‐FEP), compared with controls, by the SIFA. Representation of the regional loadings of the common factors that show significant difference between groups (two in the all FEP vs. HC, one in the S‐FEP vs. HC, and one in the M‐FEP vs. HC), in a glass brain. The loading values are reported in Table 3. Visualization with the BrainNet Viewer (http://www.nitrc.org/projects/bnv/, by Xia et al., 2013)

SIFA identified two common latent factors as different between FEP and HC; one factor in the S‐FEP versus HC comparison, and one factor in the M‐FEP versus HC comparison. The weights (sparsified loadings) of these factors are shown in Figure 3 and Table 3. The corresponding model coefficients and the 95% bootstrap confidence intervals are in Supplemental Figure S1. Common features of the latent factors were identified in each of the different comparisons (e.g., FA of body of the corpus callosum, the inferior fronto‐orbital fasciculus, and the posterior corona radiate). These common features may suggest a common pathology in FEP. Other features of the latent factors were unique to a single given population (e.g., FA in the uncinate fasciculus, the middle cerebellar peduncle, and the middle and the lateral fronto‐orbital areas, were only identified in the S‐FEP factor). These specific features may express distinctions among FEP subgroups. Within each group comparison, the SIFA factors showed a large overlap of features between FA and MD. This is consistent with the DTI properties, in which the FA and MD signals go in different directions.

**TABLE 3:** Sparsified loadings of the common factors that show significant difference between groups in the supervised integrated factor analysis (SIFA)

		rsfMRI	Loading	FA	Loading	MD	Loading	Volume	Loading
**FEP vs. HC**	**Factor 1 positive**	STG R‐thalamus R	0.166	GenuCC	0.244	SFOF R	0.287	SFO R	0.378
		STG R‐thalamus L	0.127	Cingulum R	0.209	IO R	0.142	AntCR R	0.101
		Rectus R‐ITG R	0.101	Cingulum L	0.158	SS R	0.084	SLF R	0.097
		MTGpole L‐thalamus R	0.063	BodyCC	0.097	IO L	0.063	SFO L	0.082
		Angular R‐thalamus R	0.043	MiddleCerebP R	0.095	ST R	0.061	Rectus R	0.064
		MOGL‐ThalamusR	0.043	IFOF L	0.06	Uncinate L	0.058	AntCR L	0.034
		SPG R‐thalamus R	0.028	Thalamus R	0.035	MT R	0.053		
		SPG R‐PrCu L	0.017	Cingulate L	0.022	MT L	0.04		
		SPG R‐dorsalACC R	0.014	MiddleCerebP L	0.002	Fusiform R	0.004		
		MTGpole L‐thalamus L	0.008						
	**Factor 1 negative**	PoCG L‐SMG R	−0.22	Uncinate L	−0.174	BodyCC	−0.294	IFGtr L	−0.262
		SOG L‐thalamus L	−0.071	Uncinate R	−0.144	MFO L	−0.181	SFG R	−0.208
		PrCG R‐thalamus L	−0.069	MFO R	−0.099	Thalamus L	−0.127	IFGop R	−0.142
		PrCG L‐PrCu L	−0.051	GP R	−0.095	CP L	−0.115	IFGtr R	−0.128
		SPG L‐thalamus R	−0.035	LFO L	−0.082	Thalamus R	−0.114	LFOG L	−0.108
		PrCG L‐SMG L	−0.003	PrC L	−0.062	GenuCC	−0.078	SPG R	−0.069
				PostCR L	−0.025	SpleniumCC	−0.056	IFGorb L	−0.056
				PostCR R	−0.001	MFO R	−0.054	SFG L	−0.028
						CP R	−0.018	MOG L	−0.005
								MTG R	−0.004
	**Factor 2 positive**	PCC R‐thalamus R	0.031	SupCR R	0.311	RetrolenticularIC R	0.114	SMG R	0.275
		MFOG L‐insula L	0.027	SupCR L	0.196	SLF R	0.088	SMG L	0.174
		PrCG R‐PrCu L	0.022	Insula R	0.095	Putamen L	0.085	MFGdpfc L	0.074
		PrCG R‐thalamus L	0.012	IF L	0.09	IT R	0.055	IFGtr R	0.056
		MFOG R‐insula L	0.002	PostLimbIC L	0.069	PostLimbIC L	0.049	Put L	0.049
		MTGpole L‐thalamus L	0	SLF R	0.068	Fusiform R	0.014	PoCG L	0.024
				IF R	0.061	PrCu R	0.003		
				Insula L	0.051	MFO L	0.002		
				Fusiform L	0.046				
				Caudate R	0.01				
				Cingulate R	0.073				
	**Factor 2 negative**	SPG R‐dorsalACC L	−0.373	AntLimbIC L	−0.129	Insula L	−0.4	SFO L	−0.24
		STGpole R‐put R	−0.215	SpleniumCC	−0.118	Caudate R	−0.303	SupCR L	−0.102
		Lingual R‐thalamus L	−0.208	SO R	−0.096	Insula R	−0.27	Cuneus R	−0.04
		SOG R‐lingual R	−0.052	IO L	−0.054	MiddleCerebP L	−0.03	PrCu L	−0.034
		IOGR‐ThalamusR	−0.035			Total white matter	−0.023	MTGpole L	−0.028
		PrCu L‐MTG R	−0.03			Uncinate R	−0.008	PostCR L	−0.021
		LFOGR‐InsulaR	−0.03					SOG R	−0.01
		LFOGR‐InsulaL	−0.018						
		IOGR‐ThalamusL	−0.009						
		SOGL‐ThalamusL	−0.004						
**S‐FEP vs. HC**	**Factor 2 positive**	STG pole R‐put R	0.163	BodyCC	0.07	SFOF R	0.253	SOG R	0.215
		Rectus R‐ITG R	0.154	Cingulum R	0.069	GP R	0.244	Cu R	0.161
		STG R‐thalamus R	0.119	Cingulum_L	0.044	MT L	0.195	PTR R	0.111
		SPG R‐dorsal ACC L	0.114	MiddleCerebP R	0.04	Insula L	0.107	SOG L	0.089
		SPG R‐dorsal ACC R	0.036	IFOF L	0.021	ST L	0.106	SFO L	0.08
		Lingual R‐thalamus L	0.028	IFOF R	0.001	MT R	0.105	MFG R	0.075
		MOG L‐thalamus L	0.011			ST R	0.087	MTGpole L	0.052
		PoCG R‐IOG R	0.009			SS R	0.072	SupCR L	0.042
		MFOG L‐STG pole R	0.001					Cu L	0.034
								Lingual L	0.021
								PostCR L	0.014
	**Factor 2 negative**	MFOG L‐insula L	−0.271	LFO L	−0.266	PostLimbIC L	−0.342	SS L	
		FusiformG R‐thalamus R	−0.141	LFO R	−0.189	Thalamus L	−0.179	SFG pole L	−0.154
		MFOG R‐insula L	−0.137	Uncinate L	−0.181	MFO L	−0.123	MTG R	−0.018
		PrCG R‐thalamus L	−0.06	MFO R	−0.157	Caudate L	−0.123	LFOG L	−0.115
		SOG L‐thalamus L	−0.058	SupCR L	−0.142	SF L	−0.048	Insula R	−0.087
		MFOG R‐insula R	−0.056	PostCR L	−0.098	Putamen L	−0.031	MOG L	−0.083
		PoCG L‐SPG R	−0.044	RetrolenticularIC L	−0.056	BodyCC	−0.002	SFG R	−0.076
		SOG R‐cu L	−0.031	SF R	−0.05			SMG R	−0.061
		PoCG L‐SMG L	−0.022	Rectus R	−0.047			IFG L	−0.013
				PostLimbIC L	−0.009			SFG pole R	−0.011
				Fusiform L	−0.003				
**M‐FEP vs. HC**	**Factor 1 positive**	MTG R‐thalamus L	0.21	PostCR R	0.145	EC L	0.166	Put R	0.274
		PoCG R‐thalamus L	0.078	PostThalamicRad R	0.106	Uncinate L	0.125	IFtr R	0.243
		PrCG L‐SPG L	0.051	RetrolenticularIC R	0.105	IFOF L	0.11	Put L	0.217
		PoCG L‐thalamus L	0.023	PostCR L	0.049	Putamen L	0.101	IFtr L	0.211
		PrCG R‐SPG L	0.014	IF L	0.045	AntLimbIC L	0.074	SFG R	0.1
		PoCG R‐thalamus R	0.009	SS L	0.037	InfFrontal L	0.049	Caudate L	0.043
				Fusiform L	0.025	SupCR L	0.035	IFop R	0.038
						GP L	0.002	MTGpole R	0.034
								SFG L	0.005
								BodyCC	0.004
								SFGpole R	0.002
	**Factor 1 negative**	FusiformG R‐thalamus R	−0.137	GenuCC	−0.293	IO R	−0.267	SFO R	−0.443
		SPG R‐thalamus L	−0.111	AntLimbIC L	−0.17	MO R	−0.173	SLF L	−0.1
		PoCG L‐SPG L	−0.101	BodyCC	−0.165	ST R	−0.112	SFO L	−0.044
		IOG R‐cu L	−0.085	AntCR L	−0.14	Cuneus R	−0.092	AntCR R	−0.018
		SOG R‐cu L	−0.08	AntLimbIC R	−0.064	Fusiform R	−0.082	SLF R	−0.016
		SOG L‐thalamus L	−0.079	Cinglum R	−0.047	Lingual R	−0.05	ACR L	−0.012
		MTG L‐thalamus R	−0.074	CP R	−0.008	PostThalamicRad R	−0.008	PoCG L	−0.005
		PoCG L‐SPG R	−0.069	IFOF L	−0.003				
		MFG R‐PCC L	−0.034						
		STG R‐thalamus R	−0.034						
		STGpole R‐put R	−0.021						

Abbreviations: Cortex*: SFG, MFG, IFG: superior, middle, inferior frontal gyri; IFGtr, IFGop, IFGorb: pars triangularis, opercularis, and orbitalis of IFG, MFGdpfc: dorsal prefrontal cortex; MFOG, LFOG: medium and lateral frontorbital gyri; STG, MTG, ITG: superior, middle, and inferior temporal gyri; SOG, MOG, IOG: superior, middle, and inferior occipital gyri; PrCG, PoCG: pre and post central gyri; PCC, ACC: posterior and anterior cingulate cortex; SMG: supramarginal gyrus; SPG: superior parietal gyrus; PrCu: precuneus gyrus. * For the analysis of FA and MD, the ROIs (no “G”) represent the white matter beneath the cortex. Deep white matter: IFOF: inferior frontoccipital fasciculus; ILF, SFL: inferior and superior longitudinal fasciculus; IC, EC: internal and external capsule; CP, CerebP: cerebral and cerebellar peduncle; CC: corpus callosum; CR: corona radiata, SS: sagittal stratum. Put: Putamen, GP: Globus Pallidus. Post, Ant, Sup, Inf: posterior, anterior, superior, inferior; L, R: left, right.

Among all selected features, the thalamus was the structure most consistently identified across all modalities (Figures 2 and 3). Rs‐fMRI correlations involving the thalamus and multiple cortical regions, particularly in the temporal areas, were selected in all SIFA factors within all comparisons. Thalamic FA and MD were selected in factors identified in the comparison between FEP versus HC and S‐FEP versus HC.

**FIGURE 4:**
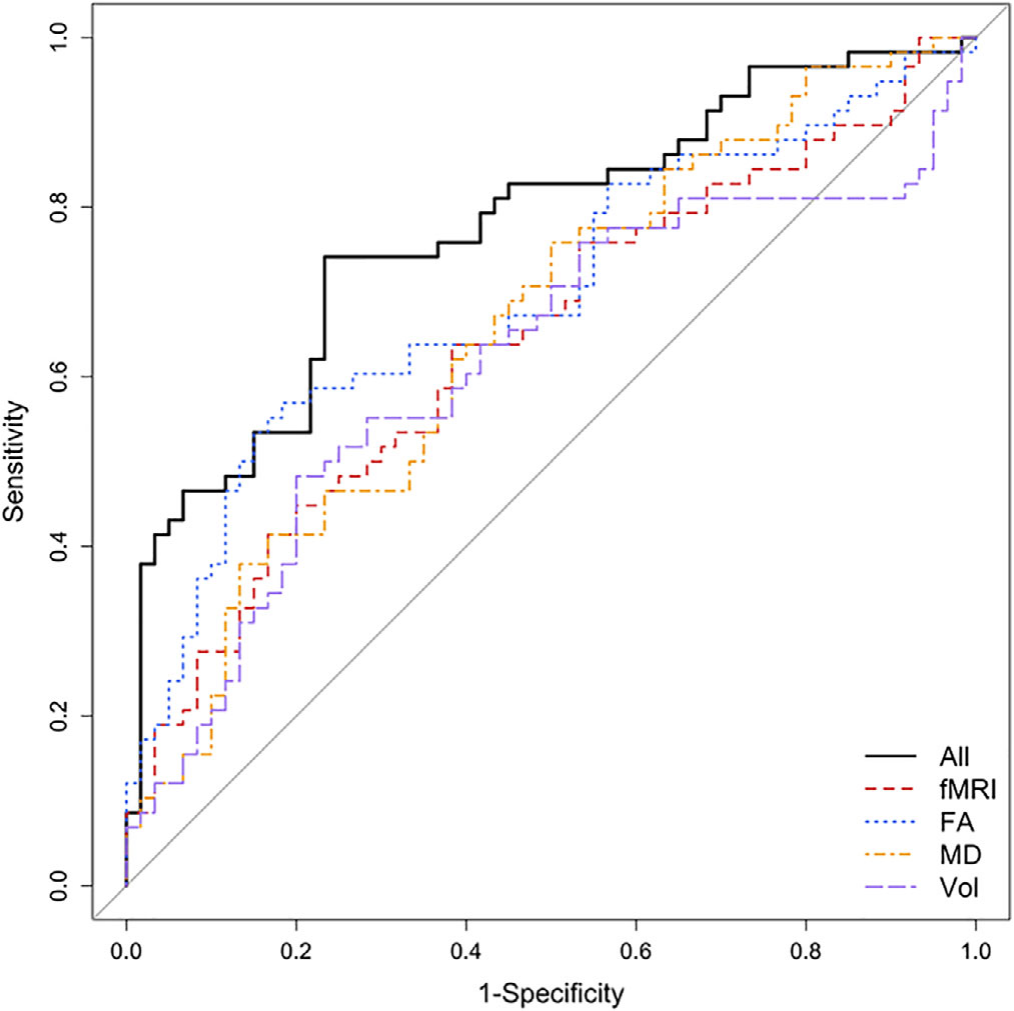
Leave‐one‐out cross‐validated ROC curve for the classification of S‐FEP and controls. The logistic models were trained using the factors (both common and individual) estimated from the SIFA. The model including multimodality‐imaging features (volumes, FA, MD, and rs‐fMRI synchrony) was the most effective on correctly classifying individuals with S‐FEP, achieving an accuracy of 77% (Table 4)

By using the SIFA factors, we accessed the power of features from singular and multiple modalities in order to classify individuals into the FEP group and its subgroups. Considering each singular modality, FA was the most effective modality in classifying the FEP groups vs. the HC group (Table 4). The integration of features from multiple modalities outperformed any singular modality used to classify S‐FEP versus HC (Figure 4). The model created with SIFA multimodality factors achieved 77% of correct classification of S‐FEP individuals, after cross‐validation. Due to the sample size and lack of comparable external dataset for testing, we used leave‐one‐out cross validation to minimize overfitting.

**TABLE 4:** Area under the curve (95% confidence interval) for the leave‐one‐out cross‐validated receiver‐operating characteristic (ROC) curves classifying of FEP and controls

	FEP vs. HC	S‐FEP vs. HC	M‐FEP vs. HC
All	0.75 (0.67–0.82)	0.77 (0.69–0.86)	0.69 (0.54–0.84)
Rs‐fMRI	0.69 (0.6–0.78)	0.64 (0.54–0.74)	0.59 (0.44–0.74)
FA	0.75 (0.67–0.83)	0.7 (0.6–0.79)	0.7 (0.57–0.84)
MD	0.64 (0.55–0.74)	0.66 (0.56–0.76)	0.6 (0.46–0.73)
T1‐volume	0.66 (0.57–0.75)	0.62 (0.51–0.72)	0.57 (0.43–0.71)

*Note:* “All” includes features from all the modalities (T1‐based volumes, DTI metrics (fractional anisotropy – FA and mean diffusivity – MD) and resting state fMRI.

Using the factors identified by SIFA yielded more robust and better performance compared with the SVM approaches. Table B S2 of the supplementary materials shows the sensitivity, specificity, and F1 score of the classification performance using the latent factors estimated from SIFA. The percentage of variation explained by the common and individual factors, as well as by all latent factors, are presented in Table B S1 of the supplementary materials. The confidence intervals of the model coefficients presented in Supplementary Figure S1 were obtained following a bootstrap procedure.

## 4 DISCUSSION

The goal of this study is to establish a pipeline that allows us to obtain meaningful brain imaging results from a relatively small sample size. We demonstrated a successful case of utilizing an unbiased, data‐driven, structure‐based analysis to characterize FEP patients. We reduced the dimensionality of the data while still preserving the individual variability, and enhanced the statistical sensitivity and power (Glasser et al., 2016). As an example, with a sample size 50 times smaller than that recently used by the ENIGMA group (27), we were able to detect similar microstructural abnormalities in DTI. The pipeline used here employs “anatomical” filters; that is, the structures in question. The definition of these structures is based on previous biological knowledge, making the interpretation of the results and possible clinical translation more straightforward (Faria et al., 2017). Using a similar set of labels for multiple modalities facilitates the combination of features derived from these modalities as well as the application of statistical methods for data fusion, such as SIFA. On the other hand, because the structure‐based analysis requires spatial pre‐definitions, it introduces challenges as the choice of parcellation and the level of granularity to be used. For example, if the phenomenon in question spatially mismatches the parcellation scheme employed, it is likely overlooked (Faria et al., 2015), a problem aggravated by the data‐driven design. Therefore, the structure‐based analysis is a complementary approach, rather than an alternative, to the voxel‐based analysis.

In the same way, SIFA is a supervised approach that facilitates the association with auxiliary covariates compared with other methodologies for dimension reduction, such as joint ICA (Moosmann, Eichele, Nordby, Hugdahl, & Calhoun, 2008). A limitation of SIFA is that the estimation procedure was derived from the normal likelihood function, which assumes the data follow Gaussian distributions. Joint ICA was designed for nonnormal data. Therefore, in our study, when implementing SIFA, proper data transformation was done to satisfy the normal assumption, for example, the functional connectivity was Fisher z‐transformed. With strong associations between the covariates and the latent structure of the multimodal imaging data, incorporating the supervised effects from covariates improves estimation accuracy and interpretability. By its particular comprehensibility and power, the combination of SIFA with the structure‐based approach is particularly relevant for translational studies, hypothesis‐generation, and for multimodal characterization of modest samples.

The power of this multimodal characterization is evidenced by the fact that multiple modality classifiers were shown to be more efficient than single modalities in classifying S‐FEP individuals. Previous MRI studies applying multivariate machine‐learning algorithms in neuroimaging have shown the potential to discriminate between individuals with schizophrenia and HCs (Cabral et al., 2016; Cetin, Houck, Vergara, Miller, & Calhoun, 2015; Du et al., 2012; Lei et al., 2020; Peruzzo, et al., 2015; Qureshi et al., 2017; Sui et al., 2013). The large range of discrimination accuracy previously reported (72–100%) is explained by the variably in samples sizes, differences in the populations and type of images analyzed, differences in validation approaches, and the “publication bias” (only the best performances are published). However, conclusions draw from patients with established schizophrenia, whose brain structure is known to be affected by the disease chronicity and long term treatment, may not less robust to FEP individuals. In fact, efficient models to discriminate chronic schizophrenic patients from HCs demonstrate poor generalizability in FEP (de Moura et al., 2018; Pinaya et al., 2016; Vieira et al., 2020). Although multimodal classifiers for FEP are rarely reported, we found that the better performance of our multimodal classifier for S‐FEP versus HCs, compared with the single modality classifiers, aligns to what was previously observed in the classification of ultra‐high‐risk individuals for psychosis, FEP, and HC (Pettersson‐Yeo et al., 2014).

In the present study, the accuracy of the classifier was increased by the combination of rs‐fMRI abnormalities, which were more specific to the S‐FEP group, with more “general” DTI abnormalities, which were greater in the S‐FEP group. This points to the value in using multimodal data integration to stratify a heterogeneous population (e.g., FEP) into subgroups of potential clinical relevance. Our group previously reported greater cognitive impairment in individuals with schizophrenia as compared with those with bipolar disorder (Schretlen et al., 2007). Other studies attempted to perform subtype prediction (Arribas, Calhoun, & Adali, 2010; Calhoun, Maciejewski, Pearlson, & Kiehl, 2008; Costafreda et al., 2011; Ota et al., 2013; Pardo et al., 2006; Rashid et al., 2016; Sacchet, Livermore, Iglesias, Glover, & Gotlib, 2015; Schnack et al., 2014; Yang et al., 2018;Schretlen et al., 2007 #1853). Although most of these studies used single modality classifiers and focused on different subgroups, they all show predictive value in modeling of “spectrum‐like” mental illness. The subgroup distinction supported by SIFA in the present study is in accordance to this notion, and adds proof of the potential value method for stratification in early disease stage.

The comprehensive characterization of the FEP population and its subgroups highlights brain areas that may represent an important locus of the pathology. One of our main findings point to widespread abnormalities in DTI (FA increase and/or MD decrease) in projection and commissural pathways. This was a very robust finding, as diverse anatomically related segments showed the same pattern, agreeing with previous findings of single modality studies in FEP (Cheung et al., 2008; Faria et al., 2019; Lyall et al., 2018; Mitelman et al., 2007; Perez‐Iglesias et al., 2010; Price et al., 2007; Schmidt et al., 2015; Wang et al., 2011; Whitford et al., 2010; Zhou et al., 2017) and in data‐driven, large sample studies of schizophrenia patients (Kelly et al., 2017; Oestreich et al., 2017). Although stronger in S‐FEP, most of the DTI features were shared in S‐FEP and M‐FEP and suggest involvement with common pathology.

In contrast to the widespread DTI features, within the functional modality we found more localized effects, and the thalamus was among the areas providing the greatest contribution to classification. The particular pattern of connectivity between the thalamus and the somato‐sensorial cortex we observed in S‐FEP aligns with observations in patients with psychotic disorders, individuals at high risk, and those in early and chronic stages of schizophrenia, as well as with reports by our group and others of structural and metabolic abnormalities centered in the thalamus (Agcaoglu et al., 2017; Altamura et al., 2017; Anticevic, 2017; Cho et al., 2016; Cho et al., 2019; Dandash, Pantelis, & Fornito, 2017; Dietsche, Kircher, & Falkenberg, 2017; Gheiratmand et al., 2017; Guo et al., 2015; Li et al., 2017; Merritt, Egerton, Kempton, Taylor, & McGuire, 2016; Murray & Anticevic, 2017; Pinault, 2017; Stephan, Friston, & Frith, 2009; Tu et al., 2019; van Erp et al., 2016; Wang et al., 2019; Woodward & Heckers, 2016; Woodward, Karbasforoushan, & Heckers, 2012; Yaesoubi et al., 2017). Note that thalamus was identified as an important structure for classification “cross‐modalities,” adding evidences to its core involvement in psychosis.

The connections between thalamus and temporal areas, and among temporal areas and basal frontal areas, were also identified as important features for classification of FEP individuals, in agreement with similar reports in patients with established schizophrenia (Lei et al., 2020). This is physiologically reasonable given the role of these areas for cognitive functions and sensory integration. Although we are tempted to draw direct correlations between brain regions and function, results from multimodal integration must be interpreted as a spatially distributed pattern rather than focusing in individual regions or features. Together, our findings indicate that both functional and physical characteristics (note that volumes of different structures were identified as important features by SIFA, despite of the lack of volumetric group differences) are implicated in FEP at the individual level.

Although our methodology is optimized for relatively modest samples, increasing the cohort would allow us to test the models in independent data, as well as cluster patients into more specific groups. A second limitation is that most of the patients were receiving psychiatric treatment at the time of the scans. The value of these findings must ultimately be proved in drug‐naïve cohorts. Information about self‐education level, handedness, disease stage, and nonantipsychotic medications was not quantitatively available; these factors were not included in our analysis. Finally, the DWI was acquired with nonisotropic voxels, which may introduce issues related to partial volume effects. Despite these limitations, it is reasonable to infer that multimodal imaging features carry information about psychosis overall, FEP subgroups and FEP individuals. The present study may serve as a proof‐of‐concept for the potential of this methodology to be used in the study of a broader range of neurological and psychiatric disorders. The combination of multiple observables within neuroimaging and across nonimage domains is crucial for conditions like FEP and most other psychiatric disorders in which there is no single dominant discriminating feature. In these cases, the subgroup and individual characterization is more likely to reside in multiple features of small effect size that capture different aspects of the condition.

## CONFLICT OF INTERESTS

S. Mori and M. I. Miller own “AnatomyWorks”. Dr. Mori is its CEO. This arrangement is managed by the Johns Hopkins University in accordance with its conflict‐of‐interest policies. All the authors have declared no biomedical financial interests or potential conflicts of interest.

## AUTHOR CONTRIBUTIONS

Andreia V. Faria: conceived, designed and performed the analysis; contributed analysis tools, wrote the paper. Yi Zhao: performed the analysis, contributed analysis tools, edited the paper. Chenfei Ye, Johnny Hsu: performed the analysis. Elizabeth Cifuentes: collected data. Lei Wang: contributed discussion. Susumu Mori, Michael Miller, Brian Caffo: contributed analysis tools. Akira Sawa: collected data, edited the paper, contributed discussion.

## Supporting information


**Supplemental Figure 1** **Estimated model coefficients (β) from supervised integrated factor analysis (SIFA)** and 95% bootstrap confidence intervals of the common factors that show difference between groups.

**Table B.1** Percentage of variation explained by the common and individual factors in each modality, as well as the percentage of variation explained by all the latent factors.
**Table B.2**. Leave‐one‐out cross‐validated sensitivity, specificity and F_1_ value of classification using the latent factors estimated from SIFA, as well as support vector machine (SVM) using linear, polynomial and radial kernels.

## Data Availability

The data analyzed in this study and the analytical code are available under request to the authors.
