# Supplementary figures and images for: Multimodal MRI assessment for first episode psychosis: A major change in the thalamus and an efficient stratification of a subgroup
Source: Hum Brain Mapp. 2020 Dec 30;42(4):1034–53. doi: 10.1002/hbm.25276 (PMC7856640; doi:10.1002/hbm.25276)

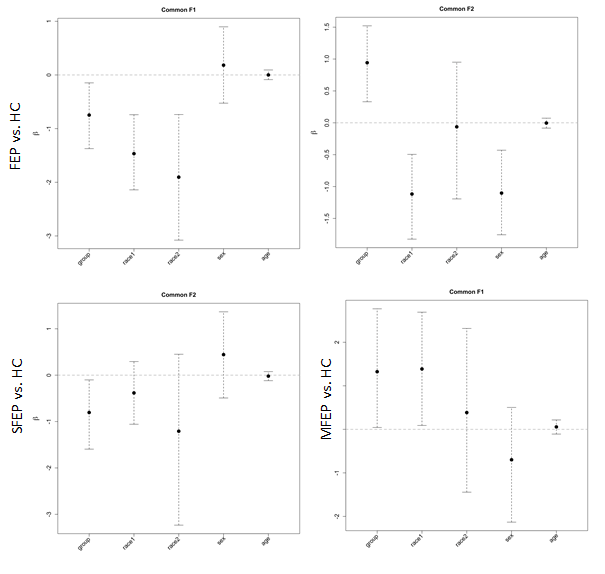

Supplement: Supplementary file 1 — Supplemental Figure 1 Estimated model coefficients (β) from supervised integrated factor analysis (SIFA) and 95% bootstrap confidence intervals of the common factors that show difference between groups. [file HBM-42-1034-s001.tif]
