# Supplementary material for: Multimodal MRI assessment for first episode psychosis: A major change in the thalamus and an efficient stratification of a subgroup
Source: Hum Brain Mapp. 2020 Dec 30;42(4):1034–53. doi: 10.1002/hbm.25276 (PMC7856640; doi:10.1002/hbm.25276)
Supplement: Supplementary file 2 — Table B.1 Percentage of variation explained by the common and individual factors in each modality, as well as the percentage of variation explained by all the latent factors. Table B.2. Leave‐one‐out cross‐validated sensitivity, specificity and F1 value of classification using the latent factors estimated from SIFA, as well as support vector machine (SVM) using linear, polynomial and radial kernels. [file HBM-42-1034-s002.docx]

**Supplementary Materials**

**Section A. Supervised integrated factor analysis (SIFA)**

We implemented the supervised integrated factor analysis (SIFA) approach to integrate data collected from multiple imaging modalities, which facilitates the characterization with auxiliary covariates (Li and Jung, 2017). Let denote the data matrix of size obtained from the th modality, for , where is the number of subjects and is the number of modalities. Let denote the covariate matrix of size , the SIFA approach assumed the following models,

, (A. 1)

, (A. 2)

, (A. 3)

, (A. 4)

, (A. 5)

where is the joint structure shared across multiple modalities, is the individual structure specific to each modality, and is the noise matrix assumed to be normally distributed. It is assumed that the joint and individual structures have low-rank decomposition. Let and denote the latent rank of the joint structure and the th modality, correspondingly. of size and of size are the latent factor matrices, and of size and of size are the corresponding loading matrices. For the latent factors, it is assumed that the factors are related to the covariates through mapping functions and , where the mapping function can be parametric or nonparametric. In the models, and are random error following multivariate normal distributions. In addition, it is assumed that , , and are mutually independent.

In this study, we assume a linear additive mapping function of the covariates. Latent factors and model parameters were estimated using an Expectation-Maximization (EM) algorithm. The ranks of the factors were chosen through an optional likelihood cross validation (LCV) method. More details of the SIFA approach are provided in Li and Jung (2017). The approach was implemented using the Matlab available at <https://github.com/reagan0323/SIFA>. To draw inference on the model parameters, a bootstrap approach was employed. The latent factors were firstly identified using the SIFA approach. Model parameters in (A.4) and (A.5) were estimated using samples by resampling with replacement. Repeat the resampling and model parameter estimation procedure for times. Bootstrap estimates and 95% confidence intervals were then obtained.

To evaluate the classification performance of the estimated latent factors, leave-one-out receiver operating characteristic (ROC) analysis was conducted. For , the data of subject was considered as the testing data and the data of the remaining subjects as the training data. A logistic regression model was fitted using the training data with the latent factors as the predictors, where sets of latent factors were considered including factors of all modalities (common and unique) and factors of each individual modality. The group of subject was then predicted by fitting the trained model on the testing set. The cross-validated area under the curve (AUC) and the 95% confidence interval were calculated. Sensitivity, specificity, and F_1_ value were also calculated at the cut-off of 0.5.

**Section B. Additional analysis results**

We present the percentage of variation explained by the common and individual factors in each modality in Table B.1. As the factors are assumed to independent, the percentage of variation explained by all latent factors is equal to the sum of the two.

**Table B.1**. Percentage of variation explained by the common and individual factors in each modality, as well as the percentage of variation explained by all the latent factors.

|  |  | **Common** | **Individual** | **All latent factors** |
| --- | --- | --- | --- | --- |
| **FEP vs. HC** | Rs-fMRI | 3.7% | 50.7% | 54.4% |
|  | FA | 4.9% | 50.1% | 55.0% |
|  | MD | 5.0% | 62.6% | 67.6% |
|  | T1-Volume | 5.3% | 65.2% | 70.6% |
| **S-FEP vs. HC** | Rs-fMRI | 4.0% | 53.1% | 57.1% |
|  | FA | 5.4% | 49.6% | 55.0% |
|  | MD | 5.4% | 62.6% | 68.1% |
|  | T1-Volume | 5.8% | 66.2% | 72.0% |
| **M-FEP vs. HC** | Rs-fMRI | 1.7% | 42.7% | 44.3% |
|  | FA | 2.3% | 43.9% | 46.1% |
|  | MD | 2.3% | 54.1% | 56.4% |
|  | T1-Volume | 2.4% | 62.2% | 64.7% |

We compared the classification performance using the factors identified by SIFA with the support vector machine (SVM) approach considering various kernel options, including linear, polynomial and radial kernel. Table B.2 presents the leave-one-out cross-validated sensitivity, specificity, and F_1_ value. In each approach, five sets of data were considered to train the prediction model, (1) data of all modalities, (2) rs-fMRI data, (3) FA values from DTI, (4) MD values from DTI, and (5) T1-volumetric data. In the SIFA approach, a logistic regression model was fitted with the estimated latent factors as the predictor. For all approaches, using all modalities improves the classification performance with more balanced sensitivity and specificity. In the FEP vs. HC study, though SVM with radial kernels attained higher sensitivity, the specificity was lower, especially when using MD from DTI and T1-volumetric data. The performance of these two approaches are similar when distinguishing S-FEP from HC. In the M-FEP vs. HC study, the SVM approach with polynomial and radial kernels failed. The SVM with linear kernel yielded higher specificity than the SIFA approach, while the sensitivity is much lower. To summary, the SIFA approach is more robust in classifying FEP patients from HC using imaging data. By integrating all modalities, the classification performance improved. Another advantage of SIFA is that it enables more straightforward interpretation of the latent factors compared to a SVM approach.

**Table B.2**. Leave-one-out cross-validated sensitivity, specificity and F_1_ value of classification using the latent factors estimated from SIFA, as well as support vector machine (SVM) using linear, polynomial and radial kernels.

|  |  |  | **SIFA** | **SVM** | | |
| --- | --- | --- | --- | --- | --- | --- |
|  |  |  |  | **Linear** | **Polynomial** | **Radial** |
| **FEP vs. HC** | All | sensitivity | 0.691 | 0.728 | 0.951 | 0.839 |
|  |  | specificity | 0.617 | 0.550 | 0.133 | 0.517 |
|  |  | F_1_ | 0.700 | 0.706 | 0.733 | 0.764 |
|  | Rs-fMRI | sensitivity | 0.728 | 0.642 | 0.901 | 0.802 |
|  |  | specificity | 0.483 | 0.533 | 0.117 | 0.550 |
|  |  | F_1_ | 0.690 | 0.646 | 0.705 | 0.751 |
|  | FA | sensitivity | 0.728 | 0.704 | 0.852 | 0.790 |
|  |  | specificity | 0.633 | 0.583 | 0.217 | 0.517 |
|  |  | F_1_ | 0.728 | 0.699 | 0.700 | 0.735 |
|  | MD | sensitivity | 0.765 | 0.691 | 0.951 | 0.802 |
|  |  | specificity | 0.467 | 0.617 | 0.150 | 0.300 |
|  |  | F_1_ | 0.708 | 0.700 | 0.737 | 0.691 |
|  | T1-Volume | sensitivity | 0.741 | 0.654 | 0.975 | 0.963 |
|  |  | specificity | 0.567 | 0.617 | 0.017 | 0.017 |
|  |  | F_1_ | 0.718 | 0.675 | 0.721 | 0.715 |
| **S-FEP vs. HC** | All | sensitivity | 0.690 | 0.621 | 0.155 | 0.621 |
|  |  | specificity | 0.777 | 0.683 | 0.950 | 0.717 |
|  |  | F_1_ | 0.714 | 0.637 | 0.559 | 0.649 |
|  | Rs-fMRI | sensitivity | 0.586 | 0.534 | 0.276 | 0.603 |
|  |  | specificity | 0.617 | 0.617 | 0.883 | 0.683 |
|  |  | F_1_ | 0.591 | 0.553 | 0.585 | 0.625 |
|  | FA | sensitivity | 0.586 | 0.621 | 0.224 | 0.552 |
|  |  | specificity | 0.767 | 0.533 | 0.983 | 0.683 |
|  |  | F_1_ | 0.641 | 0.590 | 0.610 | 0.587 |
|  | MD | sensitivity | 0.534 | 0.586 | 0.362 | 0.586 |
|  |  | specificity | 0.633 | 0.600 | 0.667 | 0.633 |
|  |  | F_1_ | 0.558 | 0.586 | 0.517 | 0.596 |
|  | T1-Volume | sensitivity | 0.603 | 0.569 | 0.069 | 0.276 |
|  |  | specificity | 0.583 | 0.617 | 0.850 | 0.533 |
|  |  | F_1_ | 0.593 | 0.579 | 0.113 | 0.314 |
| **M-FEP vs. HC** | All | sensitivity | 0.522 | 0.391 | 0.000 | 0.000 |
|  |  | specificity | 0.917 | 0.900 | 1.000 | 1.000 |
|  |  | F_1_ | 0.600 | 0.474 | 0.000 | 0.000 |
|  | Rs-fMRI | sensitivity | 0.217 | 0.435 | 0.000 | 0.000 |
|  |  | specificity | 0.933 | 0.817 | 0.950 | 1.000 |
|  |  | F_1_ | 0.312 | 0.454 | 0.000 | 0.000 |
|  | FA | sensitivity | 0.348 | 0.565 | 0.087 | 0.000 |
|  |  | specificity | 0.917 | 0.850 | 1.000 | 0.967 |
|  |  | F_1_ | 0.444 | 0.578 | 0.160 | 0.000 |
|  | MD | sensitivity | 0.130 | 0.391 | 0.043 | 0.000 |
|  |  | specificity | 0.917 | 0.783 | 1.000 | 1.000 |
|  |  | F_1_ | 0.194 | 0.400 | 0.083 | 0.000 |
|  | T1-Volume | sensitivity | 0.130 | 0.391 | 0.000 | 0.000 |
|  |  | specificity | 0.967 | 0.800 | 0.983 | 1.000 |
|  |  | F_1_ | 0.213 | 0.409 | 0.000 | 0.000 |

Li, G., Jung, S. (2017) Incorporating covariates into integrated factor analysis of multi-view data. Biometrics, 73:1433-1442.
